# Physician’s Hand-Book of Practice for 1860

**Published:** 1860-01

**Authors:** 


Physician's Hand-Book of Practice for 1860.—This little manual, compiled by Drs. Elmer and Elsberg, is again on our table. It contains a great deal of reliable information in a very small compass;—giving a brief classification of diseases, list of remedial agents, remarks on writing prescriptions, poisons and their antidotes, and a thousand little facts which an inexperienced physician might be very glad to be able to find at a moment's warning. In addition, we have room for a visiting-list, and the various memoranda required by the practitioner—making an exceedingly convenient little book. It is published by W. A. Townsend & Co., of this city.

